# Rebuttal to Correspondence on “Theoretical Threshold for Estimating the Impact of Ventilation on Materials’ Emissions”

**DOI:** 10.1021/acs.est.5c01042

**Published:** 2025-02-11

**Authors:** Fredrik Domhagen, Sarka Langer, Angela Sasic Kalagasidis

**Affiliations:** † Department of Architecture and Civil Engineering, Chalmers University of Technology, SE-41296 Gothenburg, Sweden; ‡ IVL Swedish Environmental Research Institute, P.O. Box 53021, SE-41296 Gothenburg, Sweden

We respond here to the correspondence by Deng et al. on our study.^1^ In this study, we show that the effect of increased ventilation has a negligible impact on the emission rates after the initial, near surface VOC has been emitted, which typically takes a few hours to a few days. The conclusions are drawn on the basis of available material data together with an analytical model that describes the material emission rates in a ventilated room. The simplifications made in the model are consciously chosen to give an upper limit (or worst case) for the relation between ventilation rates and emissions. Near surface resistance and room air buffering capacity (which have short-term effects on room concentration) are neglected. Also, materials are assumed to be semi-infinite, which gives an upper limit for the emission rates (materials are never depleted).

Deng et al. raise concerns that neglecting the room buffering capacity may lead to faulty conclusions regarding the effect of increased ventilation rates on emission rates. Instead, they propose an extended version of our model, which also accounts for the room buffering capacity. The proposed model in the Laplace domain is
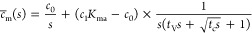
1where both *t*_c_ and *t*_V_ are time constants. Time constant *t*_c_ is described, and *t*_v_ is
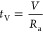
2

Deng et al. also give three solutions, depending on the relation between *t*_c_ and *t*_V_, to eq 2. Unfortunately, the solutions are not as straightforward to compute as the simple case in which the room capacity is neglected.

The question is whether accounting for the room buffering capacity is necessary for determining the effect of increased ventilation on emissions from materials. Ventilation rates in residential and office buildings are typically ≥0.5 air change per hour, which gives a *t*_V_ of ≤2 h. In other words, the relevant time scale for the room storage capacity is a few hours or less. The emission decline from new materials, on the contrary, is typically much longer, ranging from a couple of weeks to a couple of months.

As an illustration, consider a room with dimensions of 4 m × 5 m × 3 m in which all four walls consist of gypsum boards that emit VOC. The total room air volume is then 60 m^3^, and the total emitting area is 54 m^2^. Figure 1 shows a simulation of the concentration in the room using both the model proposed by Deng et al. and the model proposed by Domhagen et al.^1^ In the simulations, the emitted VOC is benzaldehyde and the following material properties are used: *D*_m_ = 3.93 × 10^–11^ m^2^/s, and *K*_ma_ = 10 053.^2^ The room is ventilated with 0.5 air change per hour, and the time constants are as follows: *t*_V_ = 2 h, and *t*_c_ = 46 h. Note that benzaldehyde diffusion in gypsum is the material–VOC combination that is identified as the most critical by Domhagen et al.^1^

**Figure 1:**
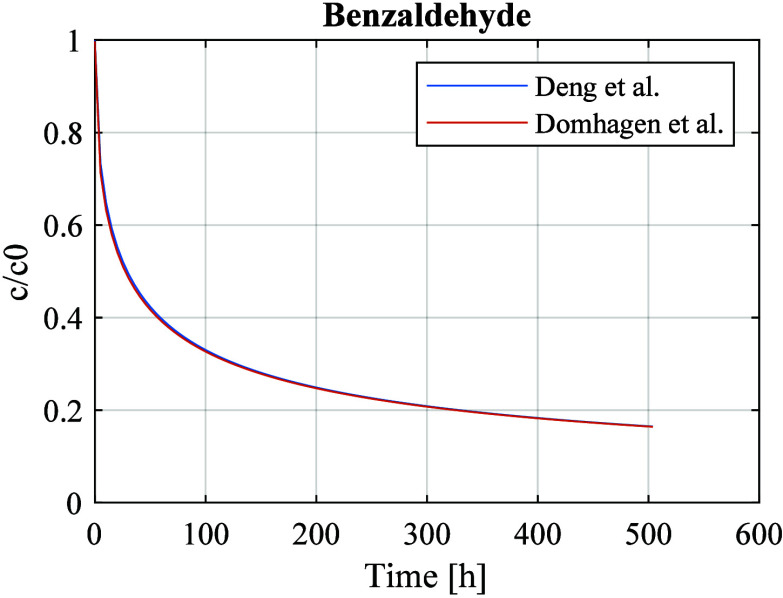
Simulations showing concentrations of benzaldehyde in a ventilated room.

Figure 2 shows the results from simulations in which ethylbenzene is emitted from the gypsum boards. The material properties are as follows: *D*_m_ = 2.15 × ^–11^ m^2^/s, and *K*_ma_ = 1550.^2^ The room is ventilated with 0.5 air change per hour, and the time constants are as follows: *t*_V_ = 2 h, and *t*_c_ = 0.6 h.

**Figure 2:**
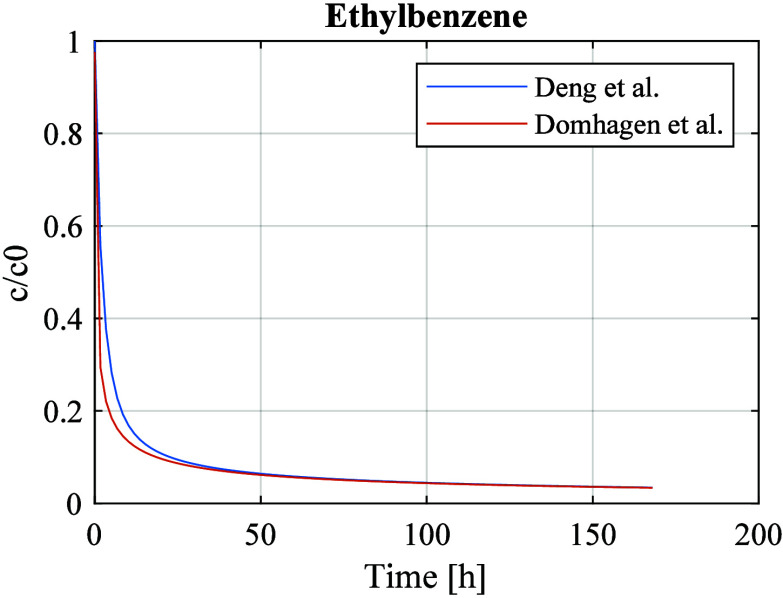
Simulations showing concentrations of ethylbenzene in a ventilated room.

Both examples illustrate that the effect of the room storage capacity has a negligible effect on the concentration at times of more than a couple of hours.

The purpose of our study was twofold: (1) to point out the existence of a ventilation threshold at which increased ventilation does not increase the rate of off-gassing of VOC and (2) to estimate an upper limit, in terms of ventilation, for such a threshold. The time scale relevant for such an analysis is a couple of days to months rather than a couple of hours, and therefore, the initial effect on the room storage capacity is not of interest.

## References

[ref1] Domhagen F.; Langer S.; Sasic Kalagasidis A. Theoretical Threshold for Estimating the Impact of Ventilation on Materials’ Emissions. Environ. Sci. Technol. 2024, 58, 5058–5067. 10.1021/acs.est.3c09815. 38445590 PMC10956430

[ref2] Yang X.; Chen Q.; Zhang J. S.; An Y.; Zeng J.; Shaw C. Y. A mass transfer model for simulating VOC sorption on building materials. Atmos. Environ. 2001, 35, 1291–1299. 10.1016/S1352-2310(00)00397-6.

